# Piloting the adaptation of the Kaufman Assessment Battery for Children—2 ^nd^ edition (KABC-II) to assess school-age neurodevelopment in rural Zimbabwe

**DOI:** 10.12688/wellcomeopenres.17902.1

**Published:** 2022-11-09

**Authors:** Joseph D. Piper, Clever Mazhanga, Gloria Mapako, Idah Mapurisa, Tsitsi Mashedze, Eunice Munyama, Marian Mwapaura, Dzivaidzo Chidhanguro, Grace Gerema, Naume V. Tavengwa, Robert Ntozini, Lisa F. Langhaug, Melanie Smuk, Tamsen Rochat, Alan Kaufman, Nadeen Kaufman, Melissa Gladstone, Elizabeth Allen, Andrew J. Prendergast

**Affiliations:** 1 Department of Genomics and Child Health, Queen Mary University of London, London, E1 4AT, UK; 2 Zvitambo Institute for Maternal and Child Health Research, Mabelreign, Harare, Zimbabwe; 3 Department of Psychology, University of the Witwatersrand, Johannesburg, 2000, South Africa; 4 Department of Medicine, Yale University, New Haven, Connecticut, 06520-8081, USA; 5 Department of Life Course and Medical Sciences, University of Liverpool, Liverpool, L69 7ZX, UK

**Keywords:** Child development, Sub-Saharan Africa, School-age, cognition

## Abstract

**Background:** Neurodevelopment assessment tools for low-resource settings are urgently needed. However, most available tools were developed in high-income settings and may lack cross-cultural validity.

**Methods:** We piloted and adapted two subtests (pattern reasoning and story completion) within the Kaufman Assessment Battery for Children-2 ^nd^ edition (KABC-II) for use in rural Zimbabwean children aged 7 years old, both within the planning domain. After initial assessments of face validity, we substituted and added items in the test battery through a co-design process with fieldworkers and child development experts. To assess how successful the changes were, T-tests adjusting for unequal variances were used to compare scores between the original and adapted versions of the same subtest. ANOVA and pairwise analysis was performed to compare the performance of KABC-II subtests across domains. Intraclass correlation coefficient was calculated to explore the variability between domains.

**Results:** Initial test scores on the planning domain were significantly lower than the other three domains of learning, sequential memory and simultaneous reasoning (P<0.001) in 50 children. Modified subtests were administered to another 20 children, who showed story completion scores that were 0.7 marks higher (95% CI 0.0, 1.4; P=0.05) and pattern reasoning scores 1.8 marks higher (95% CI 0.5, 3.2; P=0.01). Overall, the planning domain mean score increased from 8.1 (SD 2.9) to 10.6 (SD 3.4). The intra class correlation coefficient between all four KABC-II domains was initially 0.43 (95% CI 0.13, 0.64) and after modification was 0.69 (95% CI 0.37, 0.87), suggesting an increase in the construct validity.

**Conclusions:** The KABC-II planning domain was successfully adapted to improve cross-cultural validity. Construct validity was enhanced, based on increased inter-correlations among domains. This pilot has since been applied to the SHINE follow-up study. The process of co-design to modify tests for new settings may be beneficial for other commonly used neurodevelopmental tools.

## Introduction

Over 250 million children worldwide are at risk of poor cognitive development ^1^. Neurodevelopmental assessment requires tools that are adaptable to low- and middle-income (LMIC) settings. While some locally developed tools are available ^2^, many widely used tools have been developed and validated in high-resource settings, including Wechsler’s Intelligence Scales ^3^, the Bayley Scales for Infant and Toddler Development ^4^ and the second edition of the Kaufman Assessment Battery for Children (KABC-II) ^5^. These tools incorporate tests and questions that are relevant to the settings where they were developed and validated but may require a lengthy adaptation process for cross-cultural use.

**Figure 1.**
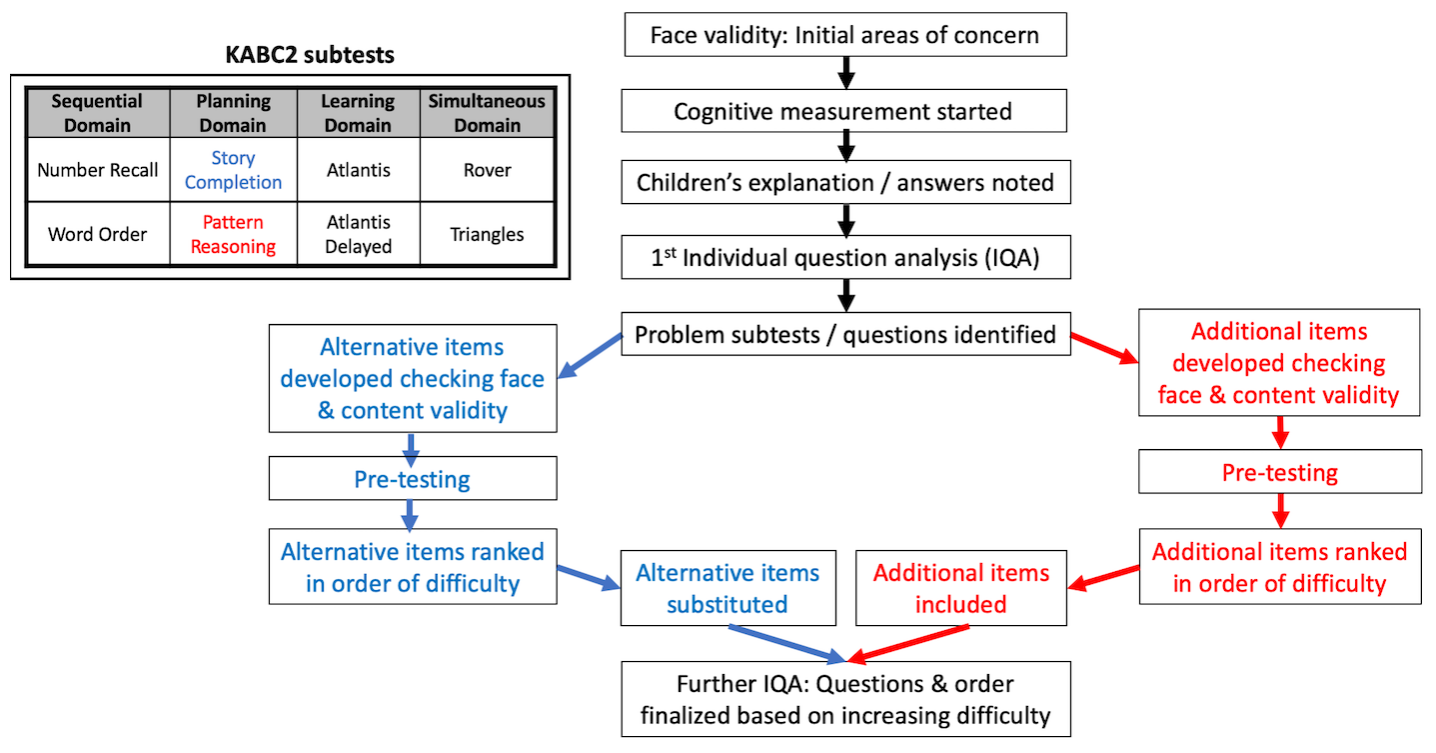
Stepwise process of monitoring and adaptation used for cognitive measurement. Inset: Structure of the KABC-II showing domains and subtests. KABC-II, Kaufman Assessment Battery for Children-2 ^nd^ edition.

One of the advantages of KABC-II is that it has a dual theoretical framework, with up to 18 individual tests (called subtests) that use either the Cattell-Horn-Carroll (CHC) psychometric model or Luria’s cognitive processing model. Luria’s approach is often used in LMICs because it measures cognitive processing by focusing on novel tests and puzzles not generally seen in schools, removing subtests that rely on acquired knowledge. This helps to correct for variable school enrolment and exposure. The KABC-II can be condensed to eight subtests and the raw subtest scores are then scaled, based on the participant’s age. The scaled results of two subtests are added together to each of the four Luria domains of cognitive processing as follows ( Figure 1) ^6^: Number Recall and Word Order subtests give the Sequential memory domain; Story Completion and Pattern Reasoning subtests provide the Planning domain; Atlantis and Atlantis Delayed subtests constitute the Learning domain; and Rover and Triangles subtests provide the Simultaneous logic domain. The subtest scaled scores can also be combined to provide the mental processing index (MPI) as an overall measure of cognitive function ^6^.

The KABC-II was originally developed and validated using a large sample of children in the USA ^5^. It has since been widely used across Africa ^7^, demonstrating robust factor analysis in Uganda ^8^ and psychometric validity and reliability in rural South Africa ^6, 9^. Recently, improved KABC-II monitoring and quality assurance has been demonstrated using regular video review ^10^ across multiple countries and languages in Africa, including with a Shona translation in Zimbabwe ^10, 11^. Using KABC-II, a significant effect on cognition was detected following a nutrition intervention in South African children aged 6–11 years old on two of the subtests, ^12^ whilst in Ethiopia, 5-year-old children with poorer growth had worse KABC-II scores than those with good growth ^13^. In Burkina Faso, five of the KABC-II subtests identified poorer scores in children with stunting ^14^. To optimally measure cognitive processing, a certain level of baseline understanding of the subtest and individual items should be achieved. Although it has been widely used across sub-Saharan Africa, there has been little documented pre-testing and piloting exploring cross-cultural validity within these individual subtests. Here we report two separate methods for piloting the adaptation of KABC-II cognitive subtests following concerns raised in their face validity during its use in rural Zimbabwe. The adaptations from this pilot study will then be applied for measuring school-aged children in rural Zimbabwe who were previously in The Sanitation Hygiene Infant Nutrition Efficacy (SHINE) cluster randomised study.

## Methods

### Participants: Study site and data collection

A pilot study to assess school-age children’s growth, physical and cognitive development was conducted in Zvamabande (rural) and Makusha (peri-urban) regions of Shurugwi district in Midlands Province, Zimbabwe between 3 ^rd^ September 2020 and 3 ^rd^ December 2020 ^15^. Inclusion criteria were children aged 7 years old who were resident in either of these two regions, identified by the Community healthworkers (CHWs) and with a primary caregiver available who was able to consent. Any children who were born into the SHINE cluster randomised trial were excluded. From the children initially identified by CHWs, 80 children were randomly selected by computer in Harare, thus minimising selection bias (80 was a convenient sample size to achieve during the three months of piloting, and enabled the team to test a variety of other measures of cognitive and physical function in the pilot study detailed elsewhere ^15^). After community sensitisation, a specific sensitisation visit to the family conducted by the CHWs using a community sensitisation sheet in Shona or Ndebele (the local languages). If the family expressed an interest in participating, a mutually convenient date was arranged. Caregivers then gave written informed consent (including the right to withdraw at any time) and children gave written assent for participation. Ethical approval for the pilot study was obtained from the Medical Research Council of Zimbabwe on 6 ^th^ April 2020 (MRCZ/A/1675). There was an amendment for this pilot study that was approved on 31 ^st^ July 2020. The main SHINE follow-up study was approved by the Medical Research Council of Zimbabwe (MRCZ), approval number MRCZ/A/1675 on 8 ^th^ February 2021. Measurements and analyses for this main follow-up study are ongoing and due to finish in 2023. Further details of the SHINE Follow-up study are available as a preprint ^16^ and on Open Science Framework.

Cognitive tests comprised the KABC-II, a school achievement test measuring literacy and numeracy, a finger-tapping task to measure fine motor skills, the caregiver-reported Strength and Difficulties Questionnaire, and a child socioemotional score, as previously described ^15^. The KABC-II was selected as part of the test battery because previous factor analysis had shown it was applicable in a similar setting in rural South Africa ^6^. KABC-II assessments were undertaken at the household (can be found as Supplementary Figure 1 in *Underlying data* ^17^). The KABC-II measurements were administered in a tent pitched close to the household, where the caregiver and child could see each other at all times.

The KABC-II measures the processing and cognitive abilities of children aged 3–18 years old. It is directly administered to one child at a time and the answers are recorded by an individual fieldworker, who has undergone extensive training, typically by a psychologist. Each subtest starts with a standardised explanation to introduce the concepts required and then sample and training questions follow. This provides the child with a standardised training for each subtest. Starting points for several subtests are based on the child’s age, with the option of dropping back to earlier starting points if an older child gets initial questions wrong. Later questions then increase in difficulty and the child stops after a discontinue rule is met, usually after getting a certain number of sequential questions wrong. Responses are recorded on a custom answer sheet to provide raw totals for each subtest, which are converted to scaled scores based on the child’s age, with younger children scoring comparatively higher for the same raw total. The eight subtests take approximately two hours to administer (including breaks).

### KABC-II adaptation

After the first 50 children were assessed, we noted that children were scoring relatively poorly on the planning domain of the KABC-II due to low scores in both story completion and pattern reasoning subtests. This observation had previously been noted in rural South Africa ^6^. We therefore decided to adapt these two subtests using a stepwise process, based on initial concerns regarding face validity, and then subsequent monitoring as cognitive measurement continued ( Figure 1). This monitoring included noting the children’s explanation of answers, and then analysing the proportion of correct answers for each individual question (individual question analysis, IQA) in both subtests for the first 50 children. This identified certain questions where unfamiliarity with the question or concept was causing poor performance. Therefore, for story completion, alternative items were developed, pre-tested and then ranked in order of difficulty before substituting problematic questions ( Figure 1). For pattern reasoning, additional items were developed, pre-tested and then ranked in order of difficulty before being included ( Figure 1). After these subtests were modified, custom scoring sheets for the raw scores were also developed, but the scaling of scores based on age was unchanged. The performance of the remaining 20 children on the modified subtests was evaluated using IQA to determine the effect of the modifications on the subtests. Permission for adaptation and translation was obtained from Pearson. We hypothesized that (a) scores would increase significantly on the two modified planning subtests, thereby providing evidence that the changes made to enhance cultural fairness were successful; and (b) correlations between domains of the KABC-II would increase after the modifications, suggesting an improvement in construct validity of the adapted test.

### Data analysis

Mean scores of the subtests for the 50 children before modification were compared to 20 children who performed the adapted subtests by using independent sample T-tests with unequal variance. One-way repeated measures analysis of variance (ANOVA) was also performed for the first 50 children to determine if the difference amongst domains was significant, with a post-hoc Tukey’s test to describe the pairwise differences between individual domains. This was similarly performed for the last 20 children. Finally, the intraclass correlation coefficient for absolute agreement using a two-way mixed effects model was calculated between domains before and after modification. Data were analysed using Microsoft Excel (RRID:SCR_016137), IBM SPSS Statistics v27 (RRID:SCR_016479) and Stata v15.0 (RRID:SCR_012763). An alternative freely available software that could perform this analysis would be R-project.

### Reflexive statement

JP is a white, male, married paediatric doctor with a foundation course in art, a medical degree from Oxford University, and diploma in tropical medicine from Liverpool University. He previously worked on body composition within the SHINE trial in a neighbouring district (Chirumanzu) in 2016. He has worked closely with many collaborators on this project (CM, IM, TM, NVT, BM, MG, AP) since 2016, which helped contribute to the co-design of the adaptations.

## Results

A total of 157 eligible children aged 7 years old were identified by CHWs, from whom 80 were randomly selected remotely by the study statistician. Among these 80 children, two families declined to be measured and three children had the wrong age on documentation, therefore five random replacements were made. Overall, 80 children (39 girls; 49%) were enrolled and underwent assessments between September 3 ^rd^ and December 4 ^th^, 2020. This was part of a broader pilot study examining growth, physical and cognitive function, which is detailed elsewhere ^15^, including baseline characteristics. Data from 10 of the 80 child assessments were used for standardisation between fieldworkers and were therefore excluded from the KABC-II adaptation process. These 10 children were all chosen during one week of standardisation measurements. Results from 70 children (35 girls; 50%) were used in the analysis of the modifications. All children successfully performed the tasks with standard instructions as recommended by the KABC-II protocol.

**Figure 2.**
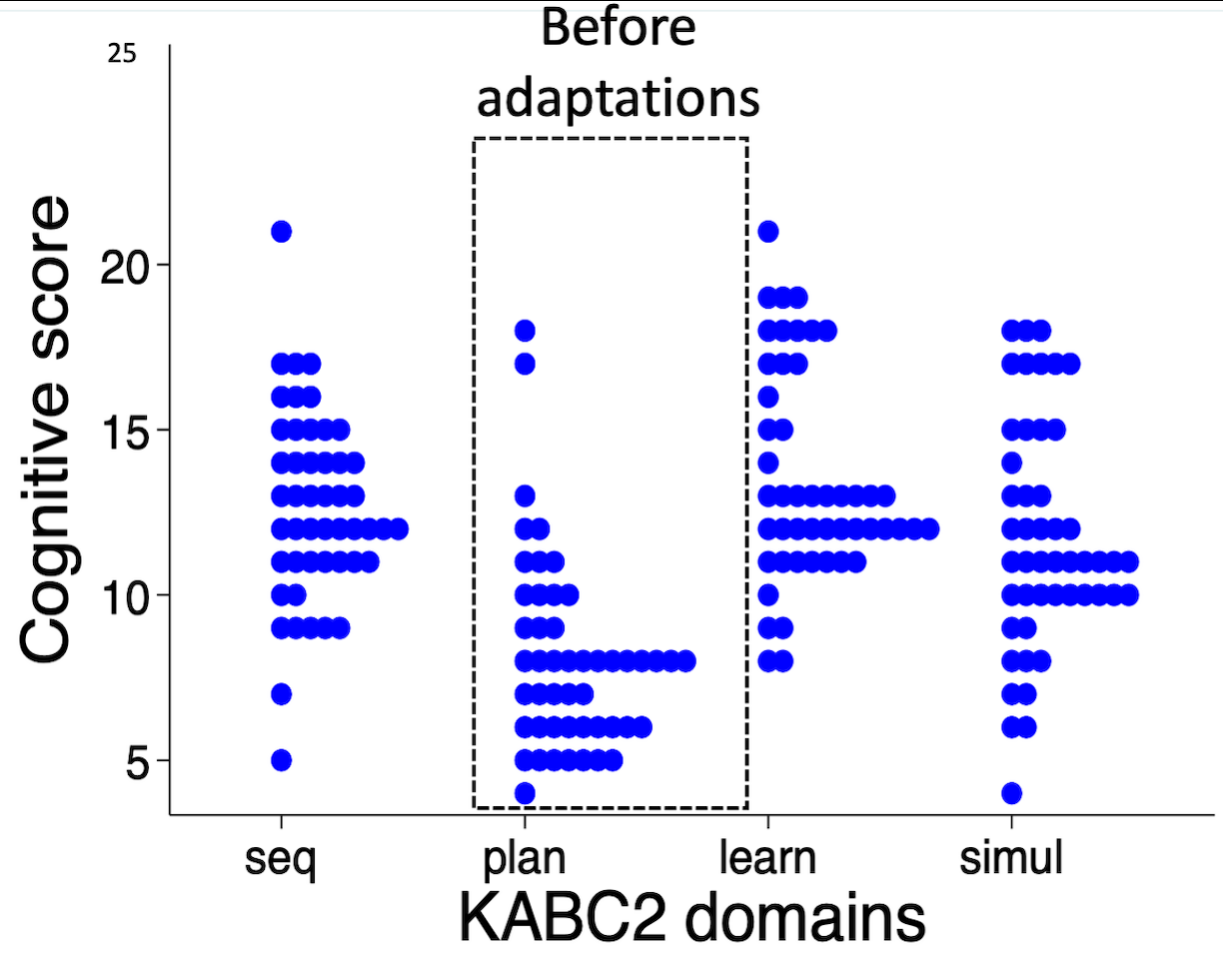
Scores for the first 50 children showed reduced scoring in the Planning domain, comprised of Story Completion and Pattern Reasoning subtests. KABC-II, Kaufman Assessment Battery for Children-2 ^nd^ edition.

Results ^17^ from the first 50 children showed that scores were significantly lower in the planning domain (mean score 8.1 (SD 2.9)), compared to the other domains (sequential 12.6 (SD 2.8), learning 13.5 (SD 3.1), simultaneous 11.6 (SD 3.5); P<0.001) ( Figure 2). A post-hoc Tukey’s test revealed that marks were significantly lower for planning compared to learning with a mean difference of 5.4 marks, (95% CI 3.8, 7.0; P<0.001), simultaneous (3.5 marks; 95% CI 1.9, 5.1; P<0.001) and sequential domains (4.6 marks; 95% CI 3.0, 6.2; P<0.001). Comparing between the other domains, the simultaneous score was less compared to learning (1.9; 95% CI 0.3, 3.5; P=0.01) but there were no other significant differences for sequential compared to learning (0.9; 95% CI -0.7, 2.5; P=0.5) or sequential compared to simultaneous (1.0; 95% CI -0.6, 2.6; P=0.34).

**Figure 3.**
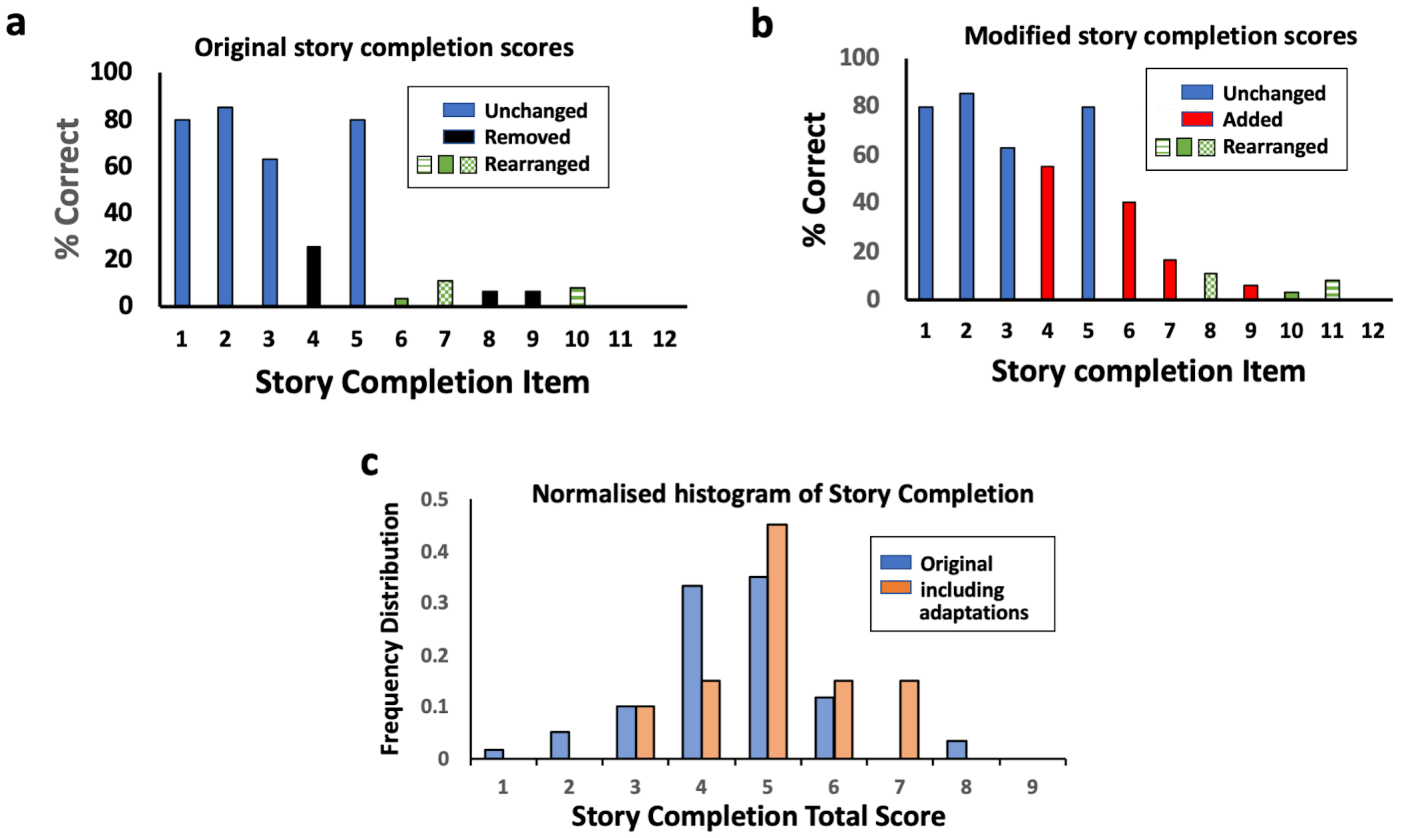
Story completion adaptation. ( **a**) Individual question analysis showed items 4, 6, 8 and 9 scored poorly. ( **b**) Adaptation of story completion showing substitutions and rearrangement of items in order of increasing difficulty. ( **c**) Normalised histogram of total scores for original and modified story completion.

The planning domain comprises two subtests: first, a story completion task, in which children have to pick the missing picture(s) from a selection of pictures, and then align them in the correct sequence to complete a picture-based story; and second, a pattern reasoning task, in which children have to select the correct printed shape or image to fit within a repeating pattern (can be found as Supplementary Figure 2–Figure 3 in *Underlying data* ^17^)

### Story completion

The story completion task contains 18 questions, although this rural Zimbabwean population of 7-year-olds did not progress past item 12. There is one sample question and three training questions, with a discontinue rule once children answer three sequential questions incorrectly. Individual question analysis identified challenges with questions 4, 6, 8 and 9 ( Figure 3a). When asked to explain their choices, it was apparent that children did not sufficiently understand the picture stories on these items to be able to complete them logically or consistently. For example, for item 4, the picture story was of a birthday cake with candles being lit, blown out and then shared. In a rural Zimbabwean context, children did not recognise the small candles on the cake and thought they were flowers, or realised that there was fire on the cake, but did not realise it was arising from birthday candles. They did not understand the sequence to blow the candles out and also did not pick consistent alternatives.

Alternative picture stories to replace items 4, 6, 8 and 9 of the story completion subtest were designed based on more familiar and locally relevant stories ( *e.g.*, washing, cooking or riding a bicycle) that could be easily described in pictures. A sequence of photographs was taken for each of the picture-based stories. These pictures were designed to match a story sequence that was similar to the KABC-II item they were replacing. They were then printed and pre-tested in a small group of eight children (can be found as Supplementary Figure 2 in *Underlying data* ^17^). However, with feedback from pretesting, the alternative stories varied in difficulty in a different way from the original sequence. For example, the new picture story of falling off a bicycle appeared easier to complete (can be found as Supplementary Figure 2 in *Underlying data* ^17^), although it was designed to replace item 9 (a picture story of a tightrope walker). We therefore re-arranged the sequence of questions so that the picture stories were administered in order of increasing difficulty, defined as the proportion of children who got them correct ( Figure 3). The proportion correct on each story completion item was calculated, both for the original story completion questions and the alternative story completion questions. Among the 20 children assessed using the modified story completion, their mean score was significantly higher (mean 4.8; 95% CI 4.2, 5.4) than among the initial 50 children assessed using only the original story completion task (mean 4.1; 95% CI 3.8, 4.4); mean difference 0.7 (95% CI 0.0, 1.4; P=0.05; Figure 3).

### Pattern reasoning

**Figure 4.**
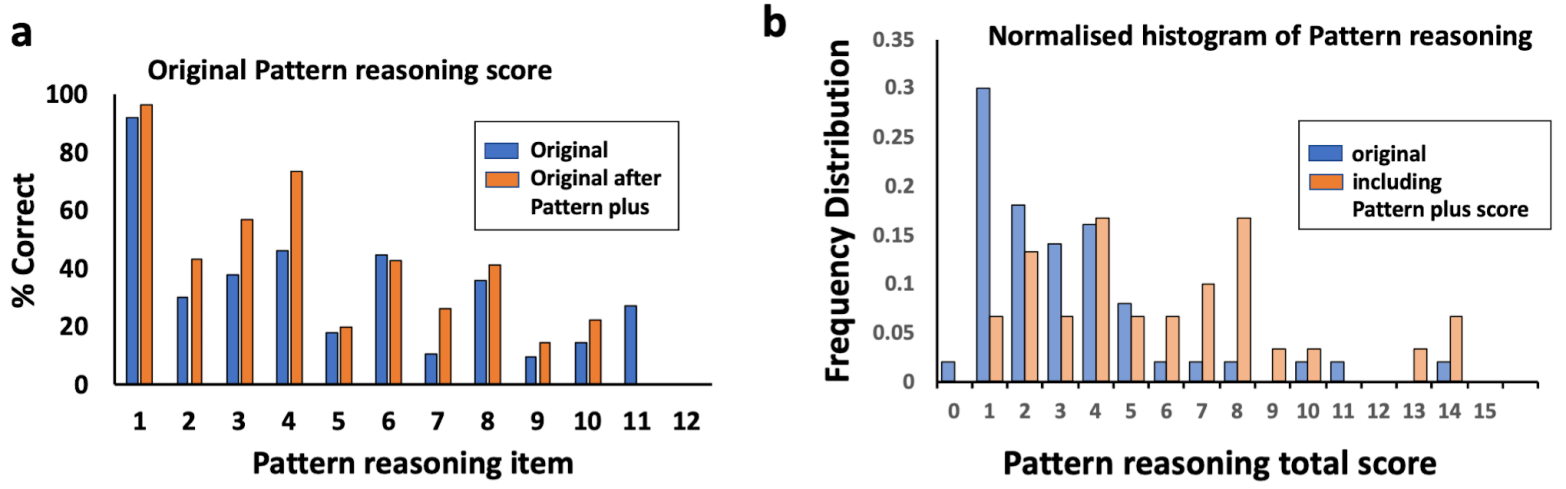
Pattern reasoning substitution. ( **a**) Scores for original KABC-II pattern reasoning items, before and after Pattern Plus training. ( **b**) Normalised histogram of total scores for original and modified pattern reasoning, including the scoring from the Pattern Plus training questions. KABC-II, Kaufman Assessment Battery for Children-2 ^nd^ edition.

Individual question analysis on the pattern reasoning subtest showed that few children were getting correct answers after item 4 ( Figure 4a). Discussion with community members and primary school teachers highlighted that these children had not previously seen puzzles with alternating patterns. The KABC-II has a single training question that demonstrates alternating patterns (item 2). It became apparent this was insufficient for children to grasp the concept consistently before answering further questions, and that they needed more examples. A total of 12 additional questions using alternating patterns were developed and then pre-tested, and the seven most appropriate questions were chosen for inclusion in the final test battery, based on face validity and feedback from the pre-testing (can be found as Supplementary Figure 3 *in Underlying data* ^17^). The selected pattern questions included shapes as well as contextually appropriate pictures of goats and chickens (can be found as Supplementary Figure 3 *in Underlying data* ^17^). All seven additional pattern questions were included as training questions for every child and included in the scoring. We termed this modified pattern reasoning “Pattern Plus” because it provided additional exposure to alternating patterns prior to the remainder of the pattern reasoning subtest. We developed routine explanations for the Pattern Plus questions to standardise the training given to the child.

For the 20 children who had these additional training questions for pattern reasoning, we first examined whether their scores had improved for the original KABC-II questions only ( Figure 4a). Their scores (mean 4.6 marks; 95% CI 3.6, 5.5) were not significantly higher than among the 50 children assessed using the original pattern completion task without training (4.0 marks; 95% CI 3.3, 4.6); mean difference 0.6 (95% CI -0.5, 1.7; P=0.3, Figure 4a). However, when the scoring from the Pattern Plus training questions was also included, the mean score significantly increased to 5.8 (95% CI 4.6, 7.0); mean difference compared to original test 1.8 (95% CI 0.5, 3.2; P=0.01) ( Figure 4b).

**Figure 5.**
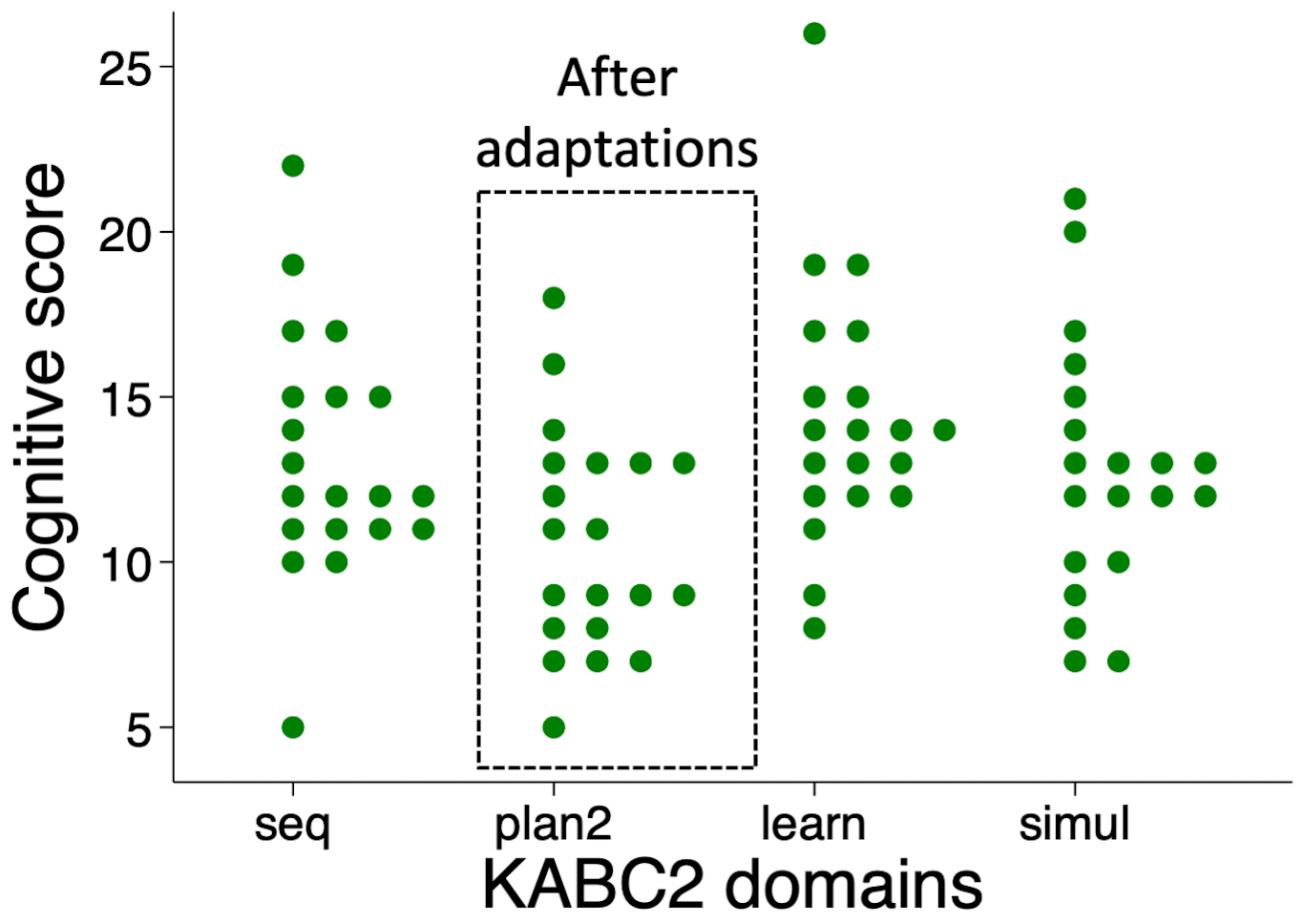
Scores for the last 20 children showed improvement in the Planning domain after modifications to the Story Completion and Pattern Reasoning subtests. KABC-II, Kaufman Assessment Battery for Children-2 ^nd^ edition.

Total scores in the planning domain among the 20 children who performed the modified story completion and pattern reasoning subtests showed a significant improvement compared to the 50 children assessed using the original KABC-II tests (mean score 10.6 (95% CI 9.0, 12.2) *versus* 8.1 (95% CI 7.2, 8.9; P=0.01), respectively; mean difference 2.5 (95% CI 0.8, 4.3; P=0.01) ( Figure 5).

When comparing domains for the last 20 children, scores still remained lower in the modified planning domain (mean score 10.6 (SD 3.4)), compared to the other domains (learning 14.3 (SD 4.0), simultaneous 12.7 (SD 3.8), sequential 13.2 (SD 3.7); P=0.001). However, a post-hoc Tukey’s test revealed that marks were significantly lower only for planning compared to learning (mean difference 3.7 marks; 95% CI 0.7, 6.8), but not for simultaneous (2.1 marks; 95% CI -0.5, 5.2) or sequential scales (2.6 marks; 95% CI -0.5, 5.7). Comparisons between the other domains showed no other significant differences for these 20 children. The intraclass correlation coefficient between domains for the first 50 children using the unmodified planning domain was 0.43 (95% CI 0.13, 0.64). For the last 20 children using the modified planning domain it was 0.69 (95% CI 0.37, 0.87).

## Discussion

There is a recognised need for context-specific tools to measure neurodevelopment in LMIC settings ^18, 19^. Many existing proprietary tools were developed in high-income settings and may not be immediately transferable to an LMIC context. We used the KABC-II to evaluate school-age cognition in rural Zimbabwe and successfully adapted two subtests using complementary methods: substitution, addition and rearrangement of items for the story completion task, and addition of further training questions for the pattern reasoning task. Both adaptations increased the scores that children achieved on these subtests, improving their overall performance in the planning domain, which was previously noted to be reduced compared to other domains in rural South Africa ^9^. The planning domain is linked to executive function, so these adaptations may improve the predictive power of the KABC-II at age 7 years old and beyond. This is important because executive functions are higher level cognitive processes needed for self-control and decision-making. They are central to healthy behaviours and development in children and adolescents ^20^. The development of executive function is highly sensitive to positive exposures such as high-quality education, and to negative exposures such as high adversity, poverty and lower-quality education ^21^. Deficits in executive functions have been associated with a wide range of negative outcomes including behavioural and mental health problems. There were no recorded harms from this study.

The increase in mean scores for both subtests (15% increase in story completion, 30% increase in pattern reasoning) suggested they became more culturally appropriate. The intraclass correlation coefficient (ICC) between domains also increased with the adaptations, suggesting variability between domains may have reduced with the modifications. The increase in ICC suggests a potential improvement in construct validity of the planning domain, with higher intercorrelations between planning and the other domains. This is supported by the confidence intervals where the ICC before modification of 0.43 (95% CI 0.13, 0.64) excludes the ICC after modification of 0.69 (95% CI 0.37, 0.87). Note, however, that the pre-modification value of 0.43 is included in the post-modification confidence interval of 0.37 to 0.87; therefore, inferences about construct validity are tentative and require cross-validation.

The outcomes of this study show the value of careful monitoring and refinement of tools for use in new settings with different contexts from their original development. Adaptation is an important process in ensuring that neurodevelopmental tests are cross-culturally applicable. Other studies have previously shown improvements in test performance with culturally relevant adaptations, for example using wire to model patterns in Zambia instead of drawing ^22^. Previously, nine different subtests from the KABC first edition (KABC) were adapted for use amongst 5–6 year olds in rural Kenya ^23^. Alterations included translation, substitution of materials including more culturally relevant pictures, as well as altering the task structures to improve cross-cultural validity. It has been proposed that adaptation of individual tests should include monitoring the distribution of scores to identify floor and ceiling effects, undertaking test and re-test reliability to ensure stable measurement, ensuring high inter-rater agreement, and comparing internal consistency with similar measures ^18^. Simultaneous measurement of contributing factors such as socioeconomic status may also demonstrate associations with variables that are expected to be related to the adapted tests (convergent validity). Repeated testing could also demonstrate increasing scores with age ^18^. Another alternative is locally developed tools such as the Malawi Developmental Assessment Tool (MDAT) ^2^ or Kilifi Development Inventory ^24^, which can then establish culturally appropriate norms. However, using these in different contexts may also require similar processes of monitoring participants’ answers and subsequent adaptation.

This study has several strengths. The process of checking face validity of items and then monitoring individual responses to questions empowered fieldworkers and the local community to monitor children’s answers and suggest adaptations, leading to a more culturally inclusive tool through co-design. The IQA also provided a way to confirm or refute initial concerns regarding face validity: for example, in the story completion task, a story about cooking a fried egg was immediately flagged and eventually replaced because rural Zimbabwean households boil or scramble eggs but do not fry them. By contrast, a series of pictures of a person blowing up balloons was well understood by children, even though the type of balloons were not commonly seen. Although scores improved, they still remained lower than for other test domains, although after modification this difference was significant only when compared to the learning domain. It is plausible that children will continue to score lower in the planning domain despite adaptation, due to cultural factors such as reduced exposure to these types of puzzle ^9^. 

The study also has several limitations. Tukey’s pairwise comparison test between domains does include an adjustment for multiple testing, but the results of comparing multiple subtests should be interpreted with some caution due to the increased risk of chance errors. For our population of 7-year-old children, no child progressed beyond item 12 on the story completion task, so our adaptation did not modify later items. Therefore, for studies using the KABC-II in older children in rural sub-Saharan Africa, we would recommend a similar monitoring phase for later items of story completion to highlight any problematic questions and then undertake pretesting of any alterations. The number of children trying each new story completion item varied, as these items became available at different timepoints. The use of photographs in story completion as an alternative to illustration may have changed some cognitive processing of the task, so ideally imaging software should be used to convert these items to illustrations. The order of the Pattern Plus sequence was decided based on increasingly complex alternating patterns, partly informed by the observation that patterns with pictures appeared more challenging than shapes. All Pattern Plus questions were asked as training questions to all the 20 children with explanations given. For young children, the addition of seven pattern plus questions may be too many, and so similar adaptations may use fewer and simpler patterns. The process of developing, printing and pre-testing alternative or additional items took time so that more children were tested before the adaptations (n=50) compared to after (n=20). Finally, test re-test reliability was not measured because it was not possible to revisit the children due to the rural locations of the measurement, although this step is recommended to demonstrate test stability ^18^.

The SHINE trial was a 2×2 factorial trial in rural Zimbabwe that was ethically approved by the Medical Research Council of Zimbabwe (MRCZ/A/1675) and registered in 2013 (NCT01824940). This trial randomized children to lipid-based nutrient supplements (LNS) and/or a comprehensive household water, sanitation and hygiene WASH intervention ^25– 28^. Mothers were enrolled in early pregnancy, with detailed data collection on home, maternal, birth and early-life factors. The adaptations from this pilot study detailed here are being applied to children born into the SHINE trial who are aged 7 years old. The SHINE follow-up study (PACTR number PACTR202201828512110) aims to measure approximately 1,300 children. Further details including the SHINE follow-up CRF’s and protocol are also available at Open Science Framework.

In conclusion, two subtests of the KABC-II were successfully adapted for use in rural Zimbabwe, drawing on both local and international expertise. This helped to increase scoring on the planning domain of cognitive processing, so that performance became more comparable to other domains in the KABC-II. The process of reviewing the face validity of items, together with monitoring of children’s responses both qualitatively and by individual question analysis helped to identify items for support. Substitution, rearrangement and addition of items can improve cross-cultural validity of cognition tools, working in collaboration with the original developers, local community and participants.

## Data Availability

Open Science Framework: Adaptation of the Kaufman Assessment Battery for Children—2nd edition (KABC-II) to assess school-age neurodevelopment in rural Zimbabwe. https://doi.org/10.17605/OSF.IO/T6GXB ^17^. This project contains the following underlying data: Extended data & results KABC-II.pdf (supplementary material) KABC_II_modification.xlsx (raw spreadsheet data of test scores) Data are available under the terms of the Creative Commons Zero "No rights reserved" data waiver (CC0 1.0 Public domain dedication).
