# Supplementary material for: Piloting the adaptation of the Kaufman Assessment Battery for Children—2 nd edition (KABC-II) to assess school-age neurodevelopment in rural Zimbabwe
Source: Wellcome Open Res. 2024 May 30;7:274. Originally published 2022 Nov 9. [Version 2] doi: 10.12688/wellcomeopenres.17902.2 (PMC11190652; doi:10.12688/wellcomeopenres.17902.2)
Supplement: Peer review history

# Reviewer report: Reviewer response for version 2

**DOI:** 10.21956/wellcomeopenres.24725.r85265

**Published:** 2024-06-20

**Authors:** Katherine Brown

**Affiliations:** 1 Great Ormond Street Hospital for Children National Health Service Foundation Trust, London, UK

Thank you. I am now happy to approve the revised article without reservations. BW Kate Brown.

Is the work clearly and accurately presented and does it cite the current literature?

Yes

If applicable, is the statistical analysis and its interpretation appropriate?

Yes

Are all the source data underlying the results available to ensure full reproducibility?

Yes

Is the study design appropriate and is the work technically sound?

Yes

Are the conclusions drawn adequately supported by the results?

Yes

Are sufficient details of methods and analysis provided to allow replication by others?

Partly

Reviewer Expertise:

I undertake health services research related to children with critical illness and with heart disease using a range of methods (data science, qualitative and health care evaluation). My interests include health care access for children with critical illness, evaluation of treatments for critically ill children, longer-term impacts of critical illness and heart diseases (neuro developmental outcomes and quality of life) and measuring experience / outcomes from the perspective of child or parent

I confirm that I have read this submission and believe that I have an appropriate level of expertise to confirm that it is of an acceptable scientific standard.

# Reviewer report: Reviewer response for version 2

**DOI:** 10.21956/wellcomeopenres.24725.r85266

**Published:** 2024-06-10

**Authors:** Supriya Bhavnani

**Affiliations:** 1 Child Development Group, Sangath, Bardez, Goa, India

My suggestions have been appropriately addressed.

Is the work clearly and accurately presented and does it cite the current literature?

Yes

If applicable, is the statistical analysis and its interpretation appropriate?

Yes

Are all the source data underlying the results available to ensure full reproducibility?

Yes

Is the study design appropriate and is the work technically sound?

Yes

Are the conclusions drawn adequately supported by the results?

Yes

Are sufficient details of methods and analysis provided to allow replication by others?

Partly

Reviewer Expertise:

Early Childhood Development, Cognitive assessments

I confirm that I have read this submission and believe that I have an appropriate level of expertise to confirm that it is of an acceptable scientific standard.

# Reviewer report: Reviewer response for version 1

**DOI:** 10.21956/wellcomeopenres.19837.r67312

**Published:** 2023-10-10

**Authors:** Katherine Brown

**Affiliations:** 1 Great Ormond Street Hospital for Children National Health Service Foundation Trust, London, UK

Katherine Brown, from the Centre for Outcomes in Children's Critical Illness and Cardiovascular Disease, University College London. 

 'Piloting the adaptation of the Kaufman Assessment Battery for Children—2 ^nd^ edition (KABC-II) to assess school-age neurodevelopment in rural Zimbabwe'  is an important, carefully planned and executed piece of work. The authors are addressing an important issue, which is that access to suitable valid and culturally appropriate measures to assess child development is not always as good as it should be. Although many measures for the assessment of child development exist, they are dominated by tools developed in high income settings, often also measures that require a very high level of training and sometimes special equipment in order for them to be used. Studies of this type are challenging to undertake and the authors have sought to carefully address an important gap within their study which has led to adaptations of the KABC-2.

 Comments and suggestions:

 Please make it clearer in the introduction whether the referenced studies 12-14 identified similar problems with the same subscales (story completion and pattern reasoning)?: these statements in introduction are not specific enough. 

 The references to the Shine study in the introduction and methods are a bit confusing. For example, in the methods the authors state that participants in Shine were excluded, but then go on to refer to the Shine follow study when discussing ethical approvals. Please clarify the relationship between this study and Shine?

 In methods, please provide a bit more detail on who undertook the assessments, their training and any additional validation or additional person review of tester activities that were undertaken. For clarity, it might be helpful to use a specific section of text on the measure and a separate section on the administration of the measure. 

 In the results section, please provide a bit more specific detail about the items that were problematic in the story completion section and pattern reasoning, which item numbers and a complete list is needed. 

 The pattern reasoning section is harder to follow than the story completion section even with reference to the supplementary materials. The rationale / reason for the substitutions is to use contextually appropriate goats and chickens, can the authors be clearer were all replaced or some, and how was this selection made? In supplementary materials pattern reasoning account, I was not able to determine exactly which patterns were original versus added. I did not find accompanying supplementary text detailing the exact reasons / issues / rationale related to the pattern reasoning section (whereas this is presented more clearly with better signposting for the story completion section again in supp materials). 

 In discussion please could the authors expand on what if any further validation work is needed for the adapted measure. It is not uncommon for test retest to be infeasible for a this type of study in a high income setting. At the same time concurrent validity / construct validity of the adapted measure could be assessed in a future study?

Is the work clearly and accurately presented and does it cite the current literature?

Yes

If applicable, is the statistical analysis and its interpretation appropriate?

Yes

Are all the source data underlying the results available to ensure full reproducibility?

Yes

Is the study design appropriate and is the work technically sound?

Yes

Are the conclusions drawn adequately supported by the results?

Yes

Are sufficient details of methods and analysis provided to allow replication by others?

Partly

Reviewer Expertise:

I undertake health services research related to children with critical illness and with heart disease using a range of methods (data science, qualitative and health care evaluation). My interests include health care access for children with critical illness, evaluation of treatments for critically ill children, longer-term impacts of critical illness and heart diseases (neuro developmental outcomes and quality of life) and measuring experience / outcomes from the perspective of child or parent

I confirm that I have read this submission and believe that I have an appropriate level of expertise to confirm that it is of an acceptable scientific standard, however I have significant reservations, as outlined above.

# Response

**Published:** 2024-05-12

**Authors:** Joseph Piper

**Affiliations:** Queen Mary, University of London, UK

Thank you for the constructive feedback. Please find responses in bold below.  

**Reviewer Comment:**

 The pattern reasoning section is harder to follow than the story completion section even with reference to the supplementary materials. The rationale / reason for the substitutions is to use contextually appropriate goats and chickens, can the authors be clearer were all replaced or some, and how was this selection made? In supplementary materials pattern reasoning account, I was not able to determine exactly which patterns were original versus added. I did not find accompanying supplementary text detailing the exact reasons / issues / rationale related to the pattern reasoning section (whereas this is presented more clearly with better signposting for the story completion section again in supp materials). 

***Author Response:***

**We apologise for the confusion. We have tried to clarify that for pattern reasoning, only further additions were made and no substitutions occurred. All of the patterns in supplementary materials were added. For copyright purposes, we have not included any of the original patterns. We have added further explanation for pattern reasoning.**

**Reviewer Comment:**

 In discussion please could the authors expand on what if any further validation work is needed for the adapted measure. It is not uncommon for test retest to be infeasible for a this type of study in a high income setting. At the same time concurrent validity / construct validity of the adapted measure could be assessed in a future study?

***Author Response:***

**Thank you for this point. This had been stated in limitations, but is now made clearer with plans for further verification within the SHINE follow-up dataset.**

# Reviewer report: Reviewer response for version 1

**DOI:** 10.21956/wellcomeopenres.19837.r53249

**Published:** 2022-12-20

**Authors:** Supriya Bhavnani

**Affiliations:** 1 Child Development Group, Sangath, Bardez, Goa, India

Having reviewed “Piloting the adaptation of the Kaufman Assessment Battery for Children—2nd edition (KABC-II) to assess school-age neurodevelopment in rural Zimbabwe”, I have found it to be an extremely interesting adaptation of the KABC-II. It is a detailed study describing the adaptation of the test, the data-driven rationale for choosing which items to adapt and piloting the adapted version on children has shown how their adaptations have improved the scale. Overall I have found this to be a well-written article. My comments are listed below –

**Abstract:**

1. In methods sentence 1, consider moving “ *both within the planning domain*” to the beginning of the sentence instead of the end, i.e. after “ *We piloted and adapted two subtests (pattern reasoning and story completion)*”. It will make for a less interrupted read.
2. Can the number of items substituted and added into each subsets of the test be mentioned somewhere in the abstract since this is one of the main results of the paper i.e. what all had to be done to the battery to make it appropriate for their settings. This could be in abstract methods or even in results.
3. Include age range or mean (sd) of the children in the methods
4. I would move the numbers of children for both phases of the pilot into the methods instead of the results in abstract

**Introduction:**

1. Expansion of LMIC is missing the word “countries”
2. Within the literature citing KABC-II use in Africa, it is unclear what subtests have worked and what haven’t in these studies in Burkina Faso, Ethiopia and South Africa. Are they also the planning domain? While I understand that adaptation and piloting for these settings have not been systematically described, could these studies be brought back into the discussion to compare how these findings compare to how KABC-II use has been described in other African settings?

**Methods:**

1. As in abstract, I suggest you mention either age range of participants of the mean and sd
2. The SHINE study appears abruptly in the methods in the sentence “Any children who were born into the SHINE cluster randomised trial were excluded.” I suggest adding in the previous sentence that the site was the same one as that in which the SHINE study was conducted.
3. In the sentence “ *After community sensitisation, a specific sensitisation visit to the family **was** conducted by the CHWs using a community sensitisation sheet in Shona or Ndebele (the local languages).*” The word “was” is missing
4. The actual process of creating the modified items for either story completion or pattern reasoning is not detailed. The word “co-design” is mentioned in some places in the manuscript but it is not clear in methods how the individual items were identified, who vetted their face validity, was Pearson involved in this process at all? Was there a committee of experts established who reviewed these items? Details of this process will enable its replication for this or indeed other developmental assessment tools in other studies

**Results:**

1. The score on the KABC-II domains before adaptation are reported in the first section. Could these be brought back into the discussion to compare them against other African studies which might have been conducted on children of similar ages?
2. Story completion section – specific items identified to be challenging mentioned here aren’t clearly different from other higher difficulty items in the graph e.g. 7, 10, 11, 12. I think the explanation is there in supplementary methods, can that please be referenced at this point so the reader knows that there is more rationale given by the authors albeit buried in supplementary data
3. Story completion section – Could the discussion comment on “why” some items of the story completion task might not have been recognizable by the children? This would help highlight the socio-cultural reasons for which the adaptation needed to be done
4. Story completion section – In the sentence “ *A sequence of photographs was taken for each of the picture-based stories.*” Please clarify that these were locally taken i.e. were African in context
5. Story completion section – In the text, please clarify where figures 3b and 3c have been referenced and not only call them figure 3.
6. Pattern reasoning section – in the sentence “ *Their scores (mean 4.6 marks; 95% CI 3.6, 5.5) were not significantly higher than among the 50 children assessed using the original pattern completion task without training (4.0 marks; 95% CI 3.3, 4.6); mean difference 0.6 (95% CI -0.5, 1.7; P=0.3, Figure 4a).*” mention that this training was Pattern Plus Training as is mentioned in the legend of Figure 4

**Discussion:**

1. Please note a few suggestions for improvement within the relevant sections above
2. The sentence “ *There were no recorded harms from this study*.” Appears out of the blue. Could it be moved to methods where the ethics are being described?
3. In the section with the sentence “ *For young children, the addition of seven pattern plus questions may be too many, and so similar adaptations may use fewer and simpler patterns.*” Could a similar comment be made on how these adaptations may or may not be sufficient for older children as well given the age range of KABC-II is 3-18 years?
4. In the sentence “ *Finally, test re-test reliability was not measured because it was not possible to revisit the children due to the rural locations of the measurement, although this step is recommended to demonstrate test stability18.*” The authors rightly comment on how not all forms of validation of the tool have been possible. I feel this needs to be expanded to include that other validations have also not been done yet, of the types that the authors themselves have mentioned in the paragraph before strengths i.e. “ *It has been proposed that adaptation of individual tests should include monitoring the distribution of scores to identify floor and ceiling effects, undertaking test and re-test reliability to ensure stable measurement, ensuring high inter-rater agreement, and comparing internal consistency with similar measures18. Simultaneous measurement of contributing factors such as socioeconomic status may also demonstrate associations with variables that are expected to be related to the adapted tests (convergent validity). Repeated testing could also demonstrate increasing scores with age18. Another alternative is locally developed tools such as the Malawi Developmental Assessment Tool (MDAT)2 or Kilifi Development Inventory24, which can then establish culturally appropriate norms. However, using these in different contexts may also require similar processes of monitoring participants’ answers and subsequent adaptation*.” – I imagine some of these will emerge from the SHINE follow up study and this can be mentioned as next steps of this work

**Figure 1:** Please include sample sizes in the flowchart at “ *cognitive measurement started*”, “ *pre-testing*” and I imagine another box would need to be added for the N=20. I feel this would make the methods flowchart more comprehensive

**Figure 2 & 5:** I wonder if it is possible to show the planning domain data as a histogram for the two conditions of before and after adaptations. It would be useful to be able to compare the results directly without looking at 2 separate figures. Plotting percentage of participants on the y axis would allow for comparison despite the different sample size.

Is the work clearly and accurately presented and does it cite the current literature?

Yes

If applicable, is the statistical analysis and its interpretation appropriate?

Yes

Are all the source data underlying the results available to ensure full reproducibility?

Yes

Is the study design appropriate and is the work technically sound?

Yes

Are the conclusions drawn adequately supported by the results?

Yes

Are sufficient details of methods and analysis provided to allow replication by others?

Partly

Reviewer Expertise:

Early Childhood Development, Cognitive assessments

I confirm that I have read this submission and believe that I have an appropriate level of expertise to confirm that it is of an acceptable scientific standard, however I have significant reservations, as outlined above.

# Response

**Published:** 2024-05-12

**Authors:** Joseph Piper

**Affiliations:** Queen Mary, University of London, UK

Thank you for the constructive feedback. Please find individual responses as below:

**Reviewer Comment:**

 Having reviewed “Piloting the adaptation of the Kaufman Assessment Battery for Children—2nd edition (KABC-II) to assess school-age neurodevelopment in rural Zimbabwe”, I have found it to be an extremely interesting adaptation of the KABC-II. It is a detailed study describing the adaptation of the test, the data-driven rationale for choosing which items to adapt and piloting the adapted version on children has shown how their adaptations have improved the scale. Overall I have found this to be a well-written article. My comments are listed below –

**Abstract:**

1. In methods sentence 1, consider moving “ *both within the planning domain*” to the beginning of the sentence instead of the end, i.e. after “ *We piloted and adapted two subtests (pattern reasoning and story completion)*”. It will make for a less interrupted read. 
***Author Response:*** **Thank you: this has been corrected.** 

1. Can the number of items substituted and added into each subsets of the test be mentioned somewhere in the abstract since this is one of the main results of the paper i.e. what all had to be done to the battery to make it appropriate for their settings. This could be in abstract methods or even in results.
***Author Response: *Thank you: This is included in abstract results**

1. Include age range or mean (sd) of the children in the methods
***Author Response: *This has been included in results so that it is clear to readers there is no difference in mean or SD in age for those before or after the modifications.  **
2. I would move the numbers of children for both phases of the pilot into the methods instead of the results in abstract
***Author Response: *We have kept the numbers of children in the results so the difference between initial and later numbers of children  in pilot is clear for the reader. **

**Reviewer Comment:**

**Introduction:**

1. Expansion of LMIC is missing the word “countries”

***Author Response: *Thank you this has been included**

**Reviewer Comment:**

1. Within the literature citing KABC-II use in Africa, it is unclear what subtests have worked and what haven’t in these studies in Burkina Faso, Ethiopia and South Africa. Are they also the planning domain? While I understand that adaptation and piloting for these settings have not been systematically described, could these studies be brought back into the discussion to compare how these findings compare to how KABC-II use has been described in other African settings?

***Author Response: *To our knowledge, examination of the suitability of KABC-II subtests has not been previously examined. We have included the studies that used all the tests to get an MPI along with those who were quoted as selecting individual subtests now in the discussion.**

**Reviewer Comment:**

**Methods:**

1. As in abstract, I suggest you mention either age range of participants of the mean and sd

***Author Response: *Thank you for this suggestion which has been included.**

  

1. The SHINE study appears abruptly in the methods in the sentence “Any children who were born into the SHINE cluster randomised trial were excluded.” I suggest adding in the previous sentence that the site was the same one as that in which the SHINE study was conducted.

***Author Response: *Thank you for this suggestion which has been included.**

  

1. In the sentence “ *After community sensitisation, a specific sensitisation visit to the family **was** conducted by the CHWs using a community sensitisation sheet in Shona or Ndebele (the local languages).*” The word “was” is missing

***Author Response: *Thank you for this correction which has been done.**

  

1. The actual process of creating the modified items for either story completion or pattern reasoning is not detailed. The word “co-design” is mentioned in some places in the manuscript but it is not clear in methods how the individual items were identified, who vetted their face validity, was Pearson involved in this process at all? Was there a committee of experts established who reviewed these items? Details of this process will enable its replication for this or indeed other developmental assessment tools in other studies

***Author Response: *We have clarified the process. Pearson were not involved apart from giving appropriate approvals, but professors Alan and Nadeen Kaufman, Tamsen Rochat and Melissa Gladstone provided appropriate input.**

**Reviewer Comment:**

**Results:**

1. The score on the KABC-II domains before adaptation are reported in the first section. Could these be brought back into the discussion to compare them against other African studies which might have been conducted on children of similar ages?

***Author Response: *Thank you for this point. Many African studies did not use the domains as specified in this paper. However, the study closest in demographics (Mitchell et al.) did and these values are now quoted with an ANOVA calculated that shows Planning is also significantly reduced.**

  

1. Story completion section – specific items identified to be challenging mentioned here aren’t clearly different from other higher difficulty items in the graph e.g. 7, 10, 11, 12. I think the explanation is there in supplementary methods, can that please be referenced at this point so the reader knows that there is more rationale given by the authors albeit buried in supplementary data

***Author Response: *Thank you for this: this has now be referenced to extended data and results in both the text and figure to assist the reader.**

  

1. Story completion section – Could the discussion comment on “why” some items of the story completion task might not have been recognizable by the children? This would help highlight the socio-cultural reasons for which the adaptation needed to be done

***Author Response: *This has also been extended in the discussion section**

  

1. Story completion section – In the sentence “ *A sequence of photographs was taken for each of the picture-based stories.*” Please clarify that these were locally taken i.e. were African in context

***Author Response: *Thank you this has been clarified**

  

1. Story completion section – In the text, please clarify where figures 3b and 3c have been referenced and not only call them figure 3.

***Author Response: *Thank you this has been clarified**

  

1. Pattern reasoning section – in the sentence “ *Their scores (mean 4.6 marks; 95% CI 3.6, 5.5) were not significantly higher than among the 50 children assessed using the original pattern completion task without training (4.0 marks; 95% CI 3.3, 4.6); mean difference 0.6 (95% CI -0.5, 1.7; P=0.3, Figure 4a).*” mention that this training was Pattern Plus Training as is mentioned in the legend of Figure 4

***Author Response: *Thank you this has been included** **Discussion:**

1. Please note a few suggestions for improvement within the relevant sections above
2. The sentence “ *There were no recorded harms from this study*.” Appears out of the blue. Could it be moved to methods where the ethics are being described?

***Author Response: *Thank you this has been added into methods**

  

1. In the section with the sentence “ *For young children, the addition of seven pattern plus questions may be too many, and so similar adaptations may use fewer and simpler patterns.*” Could a similar comment be made on how these adaptations may or may not be sufficient for older children as well given the age range of KABC-II is 3-18 years?

***Author Response: *Thank you, a comment on older children has been entered. **

1. In the sentence “ *Finally, test re-test reliability was not measured because it was not possible to revisit the children due to the rural locations of the measurement, although this step is recommended to demonstrate test stability18.*” The authors rightly comment on how not all forms of validation of the tool have been possible. I feel this needs to be expanded to include that other validations have also not been done yet, of the types that the authors themselves have mentioned in the paragraph before strengths i.e. “ *It has been proposed that adaptation of individual tests should include monitoring the distribution of scores to identify floor and ceiling effects, undertaking test and re-test reliability to ensure stable measurement, ensuring high inter-rater agreement, and comparing internal consistency with similar measures18. Simultaneous measurement of contributing factors such as socioeconomic status may also demonstrate associations with variables that are expected to be related to the adapted tests (convergent validity). Repeated testing could also demonstrate increasing scores with age18. Another alternative is locally developed tools such as the Malawi Developmental Assessment Tool (MDAT)2 or Kilifi Development Inventory24, which can then establish culturally appropriate norms. However, using these in different contexts may also require similar processes of monitoring participants’ answers and subsequent adaptation*.” – I imagine some of these will emerge from the SHINE follow up study and this can be mentioned as next steps of this work

***Author Response: *Thank you this has been included.**

**Reviewer Comment:**

**Figure 1:** Please include sample sizes in the flowchart at “ *cognitive measurement started*”, “ *pre-testing*” and I imagine another box would need to be added for the N=20. I feel this would make the methods flowchart more comprehensive.

***Author Response:*** **Thank you this has been included.**

**Figure 2 & 5:** I wonder if it is possible to show the planning domain data as a histogram for the two conditions of before and after adaptations. It would be useful to be able to compare the results directly without looking at 2 separate figures. Plotting percentage of participants on the y axis would allow for comparison despite the different sample size.  

***Author Response:*** **Thank you for this suggestion, however the histograms presented are based on individual scores and we feel this is clearer for the reader given the small numbers used in this study.**
